# Early Detection of Subclinical Atherosclerosis: Hyperhomocysteinemia as a Promising Marker in Adolescents With Vitamin B Deficiency

**DOI:** 10.7759/cureus.41571

**Published:** 2023-07-08

**Authors:** Parth S Shirode, Anjali D Parekh, Vishwa V Patel, Janmay Vala, Aniket M Jaimalani, Nilofar M Vora, Vaishnavi Gummalla, Jaineel S Patel, Nikitha Shriram

**Affiliations:** 1 Pediatrics, Terna Speciality Hospital & Research Centre, Navi Mumbai, IND; 2 Pediatrics, Surat Municipal Institute of Medical Education and Research, Surat, IND; 3 Internal Medicine, Pramukhswami Medical College, Anand, IND; 4 Internal Medicine, Terna Speciality Hospital & Research Centre, Navi Mumbai, IND; 5 Internal Medicine, Surat Municipal Institute of Medical Education and Research, Surat, IND; 6 Internal Medicine, Gandhi Institute of Technology and Management (GITAM) Institute of Medical Sciences and Research, Visakhapatnam, IND; 7 Pediatrics, Government Medical College, Surat, IND

**Keywords:** pediatric preventive medicine, homocysteine levels, hyperhomocysteinemia (hhcy), carotid intima-media thickness (cimt), pediatric clinical cardiology, endothelial injury, vitamin b deficiency, pediatrics, children and adolescents, subclinical atherosclerosis

## Abstract

In recent decades, the increased incidence of cardiovascular disease (CVD) mortality among young adults has raised concerns. Although clinical manifestations of CVD typically occur later in life, the underlying pathological processes emerge early on. This review article summarizes the association between vitamin B deficiency-induced hyperhomocysteinemia and subclinical atherosclerosis in adolescents. Numerous studies have demonstrated that elevated homocysteine levels are an independent risk factor for endothelial dysfunction (ED) and arterial stiffness, which are key contributors to CVD. Notably, vitamin B deficiency, particularly in vitamin B9 and vitamin B12, emerges as a significant factor in childhood hyperhomocysteinemia, initiating the development of subclinical atherosclerosis in early life. A comprehensive review of relevant literature from prominent bibliographic databases, including PubMed/MEDLINE, PubMed Central, Google Scholar, and Cochrane, was performed. Four cross-sectional studies focusing on homocysteine levels as an exposure variable and markers of atherosclerosis as outcome measures were included and reviewed as part of our analysis. The reviewed studies demonstrate a positive correlation between homocysteine levels and markers of atherosclerosis, including increased carotid intima-media thickness (CIMT) and ED. Mainly, adolescents with vitamin B12 deficiency exhibit a significant positive correlation between homocysteine levels and CIMT. These findings underscore the potential of hyperhomocysteinemia as an early marker for detecting subclinical atherosclerosis in adolescents with vitamin B deficiency. Despite limited research in this area, recognizing the importance of early detection and management of subclinical atherosclerosis in adolescents can help mitigate the risk of severe cardiovascular events such as myocardial infarction and stroke in young adulthood.

## Introduction and background

Cardiovascular diseases (CVDs) remain a leading cause of mortality worldwide, accounting for 32% of deaths, with over 75% occurring in developing countries [1]. Hyperhomocysteinemia, an independent major risk factor for CVDs such as atherosclerosis, myocardial infarction, and stroke, is a growing public health concern, especially among the adult population [2]. Although clinical manifestations of CVDs occur later in life, pathological processes begin early [1].

Increased homocysteine levels have solid implications for endothelial dysfunction (ED) and subsequent cardiovascular events. Hyperhomocysteinemia causes ED by various mechanisms, including disruption of the endothelial antioxidant system and increasing the intracellular concentration of reactive oxygen species, resulting in direct oxidative stress and endothelial damage. This causes impairment in lipid metabolism, further contributing to atherosclerotic lesions [3]. Deficiencies in cofactors involved in homocysteine metabolisms, such as vitamins B2 (riboflavin), B6 (pyridoxine), B9 (folic acid or folate), and B12 (cobalamin), can lead to hyperhomocysteinemia [4]. These B vitamins play a critical role in homocysteine metabolism. Specifically, vitamin B12 and vitamin B9 are necessary for converting homocysteine to methionine, while vitamin B6 is required for converting homocysteine to cysteine [4]. Various observations have been made on how higher homocysteine levels can be associated with increased vascular toxicity, including S-adenosylhomocysteine build-up due to impaired transmethylation reaction caused by vitamin B9 and B12 deficiencies [3].

Vitamin B deficiency is a common nutritional deficiency in children worldwide, with varying prevalence rates depending on the geography, dietary preferences, socio-economic class, and specific type of vitamin B. Common causes include diminished dietary intake, malabsorption syndromes, chronic diseases, worm infections, autoimmune diseases, and certain medications. Vitamin B9 and B12 deficiencies are commonly associated with elevated homocysteine levels [5]. Such vitamin B deficiency-induced hyperhomocysteinemia may contribute to subclinical atherosclerosis in childhood and adolescence, which can lead to CVDs in early adult life. Over the past two decades, there has been a significant increase in hospitalizations due to acute ischemic stroke among men and women aged 18 to 44 years old, along with a rise in acute myocardial infarction occurrences among patients under 55 years old [6]. These data warrant investigating the importance of homocysteine as an independent marker for subclinical atherosclerosis.

This review article aims to study the association between elevated homocysteine levels and increased arterial stiffness in adolescents with vitamin B deficiency-induced hyperhomocysteinemia. Despite limited research on the association between vitamin B deficiency-induced hyperhomocysteinemia and subclinical atherosclerosis in adolescents, this article will offer valuable insights into the importance of early detection and management of hyperhomocysteinemia to prevent subsequent cardiovascular events.

## Review

Search strategy

The methodology for this narrative review involved conducting a comprehensive search of various electronic databases, including PubMed/Medline, PMC Central, Google Scholar, and the Cochrane database. The search was conducted using the following keywords: "vitamin B deficiency," "vitamin B12 deficiency," "folate deficiency," "hyperhomocysteinemia," "atherosclerosis," "adolescents," and "children." These keywords were combined using the Boolean operators "AND" and "OR" to create a broad search strategy.

A mesh strategy was then applied to narrow down the search results and identify articles that discussed the relationship between elevated levels of homocysteine and subclinical atherosclerosis in adolescents. The inclusion criteria for this review consisted of case-control studies published in English, with full text available after the year 2000, involving a pediatric population (<18 years) of all ethnicities and genders, with cardiovascular risk factors as cases, and a healthy population as controls.

To ensure the quality of the studies included in this review, we excluded randomized controlled trials (RCTs), non-RCTs, cohort studies, review articles, systematic reviews, animal studies, case series, and case reports. The literature search was independently conducted by two authors, and any discrepancies were resolved by the third author.

Results

According to Zhu et al. (2006), significant correlations were observed, with notable gender differences. The associations between homocysteine, body mass index (BMI), intima medial thickness (IMT), and flow-mediated dilatation (FMD) were found in girls but not in boys. However, the correlation between homocysteine, IMT, and FMD was mediated by BMI, as indicated by nonsignificant partial correlation coefficients after controlling for BMI [7].

Erkoçoğlu et al. (2013) found that higher homocysteine levels were associated with obesity and increased carotid intima-media thickness (CIMT) in adolescents. They also highlighted a negative correlation between homocysteine levels and folic acid and vitamin B12 levels. Homocysteine was identified as an independent risk factor for CIMT, while the relationship between endothelial markers and CIMT was mostly nonsignificant, except for a potential association with plasminogen activator inhibitor-1 [8].

Akif Dundar et al. (2018) observed that children with vitamin B12 deficiency and increased homocysteine levels exhibited elevated levels of circulating CD144+EMP and CD146+EMP, which are markers of ED and vascular injury. They also concluded that elevated homocysteine showed a statistically significant positive correlation with increased CIMT, suggesting that hyperhomocysteinemia can be used as a marker for the early detection of atherosclerosis in adolescents with vitamin B12 deficiency [9].

Celik et al. (2018) reported a significant positive correlation between CIMT and homocysteine. This suggests that higher homocysteine levels are associated with increased CIMT. Multiple linear regression analysis examining the relationship between CIMT and homocysteine revealed that each 1-degree increase in homocysteine was associated with a 0.01 mm increase in CIMT (B = 0.01, t = 2.39, P < 0.05). There was also a statistically significant negative correlation between CIMT and vitamin B12 (r = -0.27, P < 0.05), indicating that as vitamin B12 levels decrease, CIMT tends to increase (Table 1) [10].

**Table 1:** Analysis of included studies CCA IMT: common carotid artery intima-media thickness; CIMT: carotid intima-media thickness; ICA IMT: internal carotid artery intima-media thickness; FMD: flow-mediated dilatation.

Reference (year)	Country	Study design	Sample size	Age (years)	Data analysis	Outcome	Outcome indicator	Main results	Association
Zhu et al. (2006) [7]	People’s Republic of China	Case-control	68	Obesity group: 11.2 ± 1.9, control group: 11.8 ± 1.5	Pearson correlation and partial correlation	Arterial stiffness	CCA IMT, ICA IMT, FMD	Correlation of homocysteine with: 1. CCA IMT: girls = 0.39 (p = 0.054); boys = 0.03 (p = 0.404). 2. ICA IMT: girls = 0.39 (p = 0.035); boys = 0.03 (p = 0.842). 3. FMD: girls = −0.40 (p = 0.031); boys = −0.04 (p = 0.773)	Positive for ICA IMT in girls; negative for FMD in girls
Erkoçoğlu et al. (2013) [8]	Ankara, Turkey	Case-control	80	Type 1 diabetes mellitus group: 14.8 ± 1.5, obesity group: 14.9 ± 1.6, obesity with glucose intolerance group: 15.0 ± 1.6, control group: 14.9 ± 1.6	Pearson’s correlation for normally distributed data. Spearman’s rank correlation for non-normally distributed data. Univariate and multivariate correlation	Arterial stiffness	CIMT	1. Pearson's correlation of homocysteine with CIMT: p = <0.001. 2. Univariate correlation of CIMT with homocysteine: 95% CI = 0.006-0.010; p = <0.001. 3. Multivariate correlation of CIMT with homocysteine: 95% CI = 0.006-0.010; p = <0.001	Positive
Akif Dundar et al. (2018) [9]	Kayseri, Turkey	Case-control	88	Vitamin B deficiency: 14.4 ± 1.72, control: 13.4 ± 1.86	Correlations by Pearson product	Arterial stiffness	CIMT	Correlation between CIMT and homocysteine: r = 0.50; p = <0.001	Positive
Celik et al. (2018) [10]	Aydin, Turkey	Case-control	100	Vitamin B12 deficient group: 14.4 ± 1.72, healthy controls: 13.4 ± 1.86	Multilinear regression analysis	Arterial stiffness	CIMT	Correlation of CIMT with homocysteine: r = 0.37; p = <0.001	Positive

Apart from the above-mentioned studies, a study conducted by Monasso et al. (2021) suggests that concentrations of circulating total vitamin B12, vitamin B9, and homocysteine during fetal life appear to be associated with markers of subclinical atherosclerosis during school age. As compared to normal early-pregnancy serum total vitamin B12 concentrations (145 pmol/L) and plasma vitamin B9 concentrations (8 nmol/L), low serum total vitamin B12 concentrations (<145 pmol/L) and low plasma vitamin B9 concentrations (<8 nmol/L) were associated with higher CIMT and lower carotid distensibility, respectively, in the children of school age [11]. One standard deviation score (SDS) higher plasma homocysteine concentrations measured in cord blood at birth was associated with a 0.05 SDS (95% CI: 0.09, 0.02) lower carotid distensibility at school age [11].

Discussion

Over 50 years ago, a study conducted by McCully (1969) linked increased homocysteine levels with the development of coronary vascular disease. His initial observation stemmed from two distinct case studies involving children who died as a result of rare genetic conditions: one related to homocystinuria caused by abnormal cobalamin (vitamin B12) metabolism, and the other associated with homocystinuria caused by cystathionine B-synthase deficiency. McCully proposed that both conditions shared a common factor of elevated homocysteine, which in turn led to widespread arteriosclerotic lesions [12]. Based on this foundation, Selhub (2006) established a connection between hyperhomocysteinemia and deficiencies in vitamins B12, B6, and B9 [13].

Vitamin B and Homocysteine Metabolism

**Figure 1:**
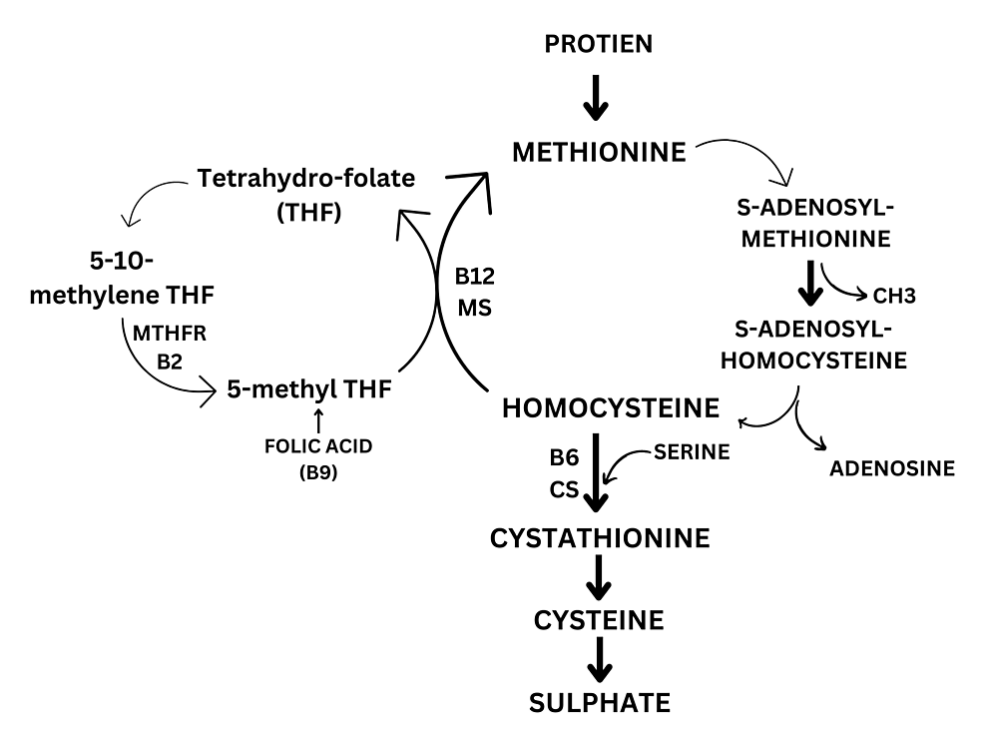
Role of vitamin B in homocysteine metabolism This figure explains the role of vitamins B2, B6, B9 (folic acid), and B12 in homocysteine metabolism. Homocysteine receives a methyl group in the remethylation pathway primarily from the transformation of 5-methyl-THF into tetrahydrofolate (THF) [15]. This B12-dependent reaction requires the enzyme methionine synthase (MS). Homocysteine condenses with serine to form cystathionine in the transsulfuration pathway, catalyzed by the cystathionine synthase (CS), which requires vitamin B6 as a cofactor [15]. 5-methylene-THF is converted to 5-methyl THF, the reaction requires the enzyme methyl tetrahydrofolate reductase (MTHFR) and vitamin B2 as cofactor. MS: methionine synthase; THF: tetrahydrofolate; MTHFR: methyl tetrahydrofolate reductase; CS: cystathionine synthase; B12: vitamin B12; B6: vitamin B6; B2: vitamin B2; CH3: methyl group. Image credits: Parth S. Shirode

Vitamin B deficiency can cause hyperhomocysteinemia [4] through its impact on the metabolism of homocysteine, an amino acid produced as a byproduct of the body's normal metabolism of methionine, another amino acid. The B vitamins, particularly vitamins B12, B6, and B9, play a critical role in the metabolism of homocysteine. Specifically, vitamin B12 and vitamin B9 are necessary for converting homocysteine to methionine, while vitamin B6 is required for converting homocysteine to cysteine. Vitamin B2 is also necessary for the conversion of methylene tetrahydrofolate (THF) to methyl THF [4] (Figure 1). These processes collectively help maintain appropriate levels of homocysteine in the body and prevent the negative health consequences associated with elevated homocysteine levels. When there is a deficiency in any of these B vitamins, the metabolism of homocysteine is impaired, leading to an accumulation of homocysteine in the blood, known as hyperhomocysteinemia. This condition can have adverse effects on cardiovascular health since high levels of homocysteine have been linked to an increased risk of heart disease, stroke, and other vascular disorders [13,14]. Therefore, ensuring an adequate intake of B vitamins through a balanced diet or supplementation is crucial for maintaining cardiovascular health [14].

Causes of Vitamin B Deficiency in Adolescents

Elevated levels of homocysteine may be attributed to nutritional deficiencies of vitamins B9, B12, B6, and B2, which are major enzyme cofactors required for normal homocysteine metabolism.

Vitamin B9 (folic acid): Diminished dietary intake (e.g., fussy or uncooperative children and picky eaters who are very selective with their dietary preferences), individuals deficient in enzymes involved in B9 metabolism, heat exposure during cooking, diseases affecting the jejunum (where B9 is absorbed), such as celiac disease, small bowel syndrome, and tropical sprue, and intake of certain medications for conditions like seizures (e.g., phenytoin) and autoimmune disorders (e.g., methotrexate), have all been known to be associated with folic acid deficiencies. Hence, children and adolescents with seizure disorders and autoimmune disorders are at a higher risk of developing these deficiencies. Folic acid deficiencies may also occur following a deficiency of vitamin B12, which can be attributed to the folate trap phenomenon [16]. This increases urinary folate excretion. Additionally, hemolytic anemia, pregnancy, and dialysis can lead to folate deficiency [16].

Vitamin B6: Isolated vitamin B6 deficiency is highly unusual. Insufficient levels of vitamin B6 typically coincide with decreased levels of other B-complex vitamins, including vitamins B12 and B9. As the deficiency of vitamin B6 progresses, noticeable biochemical changes start to occur. Some causes of vitamin B6 deficiency are disorders affecting the kidneys (such as chronic kidney disease) and the lower gastrointestinal system (such as celiac disease and inflammatory bowel disease). Long-term use of antiepileptic drugs (as seen in children with seizure disorders) can also cause B6 deficiency [17].

Vitamin B12: Following a vegetarian diet is one of the major causes of vitamin B12 deficiency because a diet high in meat is the primary source of B12 [18]. Other causes include gastrointestinal pathologies (including *Helicobacter pylori* infection, tropical sprue, and hypochlorhydria) or those who have had gastric surgeries. If the vitamin B12 deficiency is not identified and treated, infants breastfed by vitamin B12-deficient mothers are particularly vulnerable to possible fatal consequences [19]. In addition to this, autoimmune metaplastic atrophic gastritis (which causes pernicious anemia) may also lead to B12 deficiencies [20].

Vitamin B2 (riboflavin): Insufficient consumption of riboflavin or hormonal imbalances can lead to a deficiency in this nutrient. Additionally, riboflavin deficiency is associated with other B complex vitamins. Pregnant and breastfeeding women, individuals diagnosed with Brown-Vialetto-Van Laere syndrome, and those following a vegan diet are also susceptible to developing riboflavin deficiency [21].

Prevalence of Vitamin B Deficiency

Areas around the world with increased risk of having lower vitamin B12 intake include the Indian subcontinent (80% of Indian preschoolers between the ages of three to five years [22], and 70% of Indian adults [23]), the regions of Central and South America, and some regions within Africa [24] (70% in Kenyan school children [25,26]). It was found that individuals with ancestral ties to India and Pakistan residing in Toronto exhibit a substantial prevalence of vitamin B12 deficiency, which can be linked to lower consumption of meat and dairy products when compared with deficiency rates throughout the rest of the population [27].

A cross-sectional study conducted by Chakraborty et al. (2018)* *among 2403 school-going children and adolescents in Haryana, India uncovered that the occurrence of vitamin B12 deficiency was 32.4% (43.9% in rural areas and 30.1% in urban areas; 34.4% of them were male and 31.0% of them were female; 28.1%, 39.8%, and 51.2% of them were of normal BMI, overweight, and obese, respectively) [28]. The majority of the obese adolescents (51.2%) were vitamin B12 deficient. Age took precedence over some other factors in vitamin B12 deficiency among rural adolescents, whereas BMI was associated with serum B12 levels in urban adolescents [28]. Rural female and urban male populations have lower serum vitamin B12 levels in contrast with their respective coevals [28].

In another study conducted by Wong et al. (2021) involving preschool children in Guatemala, the national occurrence of vitamin B12 deficiency in children was found to be 22.5% and that of B9 deficiency in children was found to be 33.5% [29]. A study by Kapil et al. (2014) concluded that in high-income, middle-income, and low-income groups of adolescents (between the ages of 11-18 years in Delhi, India), the prevalence of folate deficiency was 22.5%, 40.4%, and 52.2%, respectively [30]. These data allude to the fact that folate deficiency is much more common among adolescents belonging to the low-income groups as compared to those who fall into the high-income groups [30].

Vitamin B12 status in the United States has been previously assessed in the National Health and Nutrition Examination Survey. Based on this data, low B12 levels rose as individuals advanced in age and were generally more prevalent among women than men (prevalence of 3.3% vs. 2.4%) [31]. A substantial occurrence of low B12 levels is not limited to older adults, as certain countries report prevalence rates exceeding 40% among various subpopulations, such as children, emerging adults, women in their reproductive years, expectant mothers, and other adults [32].

Although vitamin B12 and vitamin B9 deficiencies are most commonly associated with hyperhomocysteinemia, vitamin B6 deficiency has also demonstrated some correlation but isolated vitamin B6 deficiencies are slightly unusual. During the analysis of the 2003-2004 National Health and Nutrition Examination Survey data (in the United States), it was observed that concentrations of vitamin B6 remained low in certain groups despite their intake of 2.0-2.9 mg/day, which exceeds the current recommended dietary allowance. The study evidenced that teenagers displayed the lowest vitamin B6 concentrations, followed by adults aged 21-44 years among the population chosen [17,33].

Riboflavin deficiency is a highly uncommon condition in the United States [21]. However, it is prevalent in developing countries in Asia and Africa. Older adults, individuals with alcohol addiction, and women using birth control pills are at a higher risk of developing riboflavin deficiency because the body's ability to absorb riboflavin is limited while taking birth control pills [21].

Homocysteine and Atherosclerosis

Homocysteine levels exceeding 15 μM/L in plasma are categorized as moderate (15-30 μM/L), intermediate (30-100 μM/L), or severe (>100 μM/L), with the threshold set at 15 μM/L [34]. Plasma homocysteine levels greater than 15 μM/L have been associated with a mortality rate of 24.7%, compared to 3.8% in individuals with levels below 9 μM/L [35]. Elevated levels of homocysteine are associated with an increased risk of hypertension, coronary artery disease, myocardial infarction, and strokes [36]. Studies have demonstrated that homocysteine stimulates thrombosis through various mechanisms. It promotes the production of thromboxane A2, which acts as a vasoconstrictor and pro-aggregant in platelets [37]. Additionally, homocysteine indirectly inactivates the anticoagulant proteins (protein C and thrombomodulin), activates the procoagulant endothelial cell factor V, and enhances thrombin production [37].

Homocysteine can impair the endothelium’s capacity to regulate vascular tone by decreasing the bioavailability of nitric oxide (NO) [38]. Also, homocysteine has a positive correlation with endothelin-1 (ET-1), a potent vasoconstrictor [39]. Homocysteine promotes the production of pro-inflammatory cytokines, such as monocyte chemoattractant protein-1 (MCP-1) and interleukin-8 (IL-8), by activating nuclear factor-kappa B (NF-κB) [40]. Additionally, it contributes to atherogenesis by inducing apoptotic cell death in endothelial and smooth muscle cells [3]. Hyperhomocysteinemia also reduces high-density lipoprotein (HDL), impairing reverse cholesterol transfer, which may further contribute to atherosclerosis [3].

**Figure 2:**
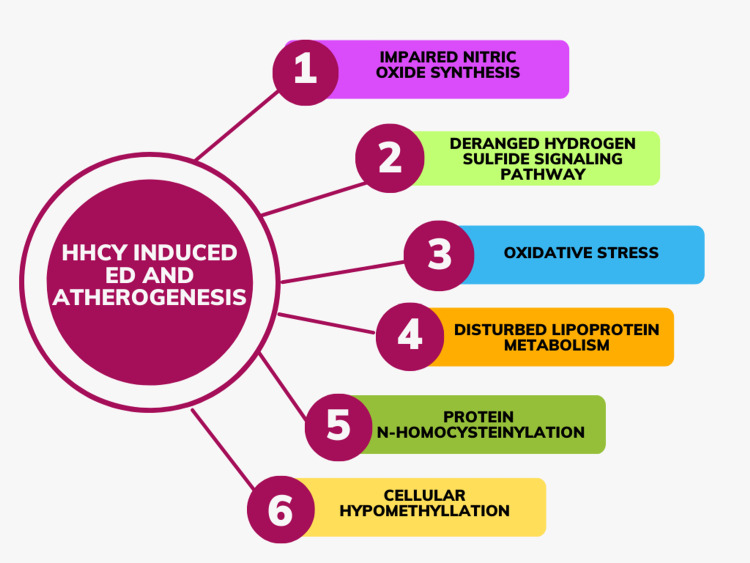
Mechanisms explaining the role of hyperhomocysteinemia (HHCY) in endothelial dysfunction (ED) and atherogenesis Image credits: Parth S. Shirode

Six mechanisms have been suggested to explain hyperhomocysteinemia-induced ED and atherogenesis: (1) impairment of nitric oxide synthesis, (2) deregulation of the hydrogen sulfide signaling pathway, (3) oxidative stress, (4) disturbances in lipoprotein metabolism, (5) protein N-homocysteinylation, and (6) cellular hypomethylation (Figure 2) [3].

Importance of Diagnosing Subclinical Atherosclerosis in Adolescents

Atherosclerosis is a chronic medical condition characterized by a long, initial, asymptomatic phase, which can ultimately progress to acute coronary events and increase the risk of stroke [41]. Clinical and epidemiologic research has shown that atherosclerosis can begin in childhood, continue through adolescence, and result in CVD by middle life. According to the Pathobiological Determinants of Atherosclerosis in Youth (PDAY) research, advanced atherosclerosis can begin in late adolescence, with the advancement of atherosclerotic plaque in response to CVD risk factors taking place in the third and fourth decades of life [6].

Although CVD mortality in the United States has decreased overall for the population since 1968, the percentage of acute myocardial infarctions attributed to individuals under the age of 55 years has grown from 27% to 32% during the last two decades [6]. Approximately 10% to 15% of all strokes occur in young adults, affecting about 2 million young adults every year. Acute ischemic stroke hospitalizations have also increased significantly [42]. This calls for measures for early screening of atherosclerosis in adolescents and young adults. To optimize therapy and lower recurrence, it is essential to identify the causes and risk factors of ischemic stroke in young people as soon as possible. Thus, early diagnosis of subclinical atherosclerosis amongst adolescents can help prevent the risks of developing severe cardiovascular and cerebrovascular diseases, such as myocardial infarction, sudden cardiac death, and stroke.

## Conclusions

The increasing incidence of cardiovascular mortality in adolescents has prompted research aimed at understanding the implicated risk factors and emphasizing early screening for prevention. Hyperhomocysteinemia, associated with deficiencies in vitamin B6, vitamin B9, and vitamin B12, is now recognized as an independent risk factor for ED and atherosclerosis. Our review of four cross-sectional studies shows a positive correlation between hyperhomocysteinemia and CIMT in vitamin B-deficient adolescents. Furthermore, the review highlighted a strong negative correlation between vitamin B and homocysteine. These findings underscore the importance of addressing vitamin B deficiencies in adolescents to prevent CVDs in young adults. However, further longitudinal and robust studies are needed to understand better the relationship between vitamin B deficiency-induced higher homocysteine levels and subclinical atherosclerosis in children and adolescents. Also, large-scale interventions are necessary incorporating ample vitamin B complex supplementation in children, with the aim of assessing their potential in preventing subclinical atherosclerosis in adolescents and promoting a healthy cardiac life.
